# Optimal treatment of adrenocortical carcinoma with mitotane: results in a consecutive series of 96 patients.

**DOI:** 10.1038/bjc.1994.183

**Published:** 1994-05

**Authors:** H. R. Haak, J. Hermans, C. J. van de Velde, E. G. Lentjes, B. M. Goslings, G. J. Fleuren, H. M. Krans

**Affiliations:** Department of Endocrinology, University Hospital Leiden, The Netherlands.

## Abstract

Mitotane is considered to be the drug of choice for patients with inoperable, recurrent and metastatic adrenocortical carcinoma, although a favourable effect of this drug on survival has never been documented. We evaluated the efficacy of mitotane treatment of 96 patients with adrenocortical carcinoma followed up in our department between 1959 and 1992. Complete tumour resection was the goal of the initial treatment. Mitotane treatment was classified according to serum trough concentrations on maintenance therapy: low (< 14 mg l-1) or high (> or = 14 mg l-1). Total tumour resection was feasible in 47 patients (49%), and subtotal resection was performed in 37 patients (39%). Patients who underwent total tumour resection survived significantly longer than those who did not (P < 0.001). Adjuvant mitotane therapy (n = 11) did not influence survival after total resection. Sixty-two patients were given mitotane treatment at some time during their illness, only 30 of whom reached high maintenance serum levels. Mitotane treatment with high serum levels had an independently favourable influence on patient survival, using univariate (P < 0.01) and multivariate analysis (P = 0.01). Mitotane treatment resulting in low serum levels was tantamount to not giving mitotane at all. We conclude that mitotane treatment in adrenocortical carcinoma is effective only when high serum levels can be achieved.


Br. J. Cancer (1994), 69, 947-951 © Macmillan Press Ltd., 1994

### Optimal treatment of adrenocortical carcinoma with mitotane: results in a consecutive series of 96 patients

H.R. Haak[1,2], J. Hermans[3], C.J.H. van de Velde[4], E.G.W.M. Lentjes[5], B.M. Goslings[1], G.-J. Fleuren[6] & H.M.J. Krans[1]

[1]*Department of Endocrinology, University Hospital Leiden, PO Box 9600, 2300 RC Leiden, The Netherlands;* [2]*Department of Internal Medicine, Diaconessenhuis, PO Box 90.052, 5600 PD Eindhoven, The Netherlands; Departments of* [3]*Medical Statistics,* [4]*Surgery,* [5]*Clinical Chemistry and* [6]*Pathology, University Hospital Leiden, PO Box 9600, 2300 RC Leiden, The Netherlands.*

**Summary** Mitotane is considered to be the drug of choice for patients with inoperable, recurrent and metastatic adrenocortical carcinoma, although a favourable effect of this drug on survival has never been documented. We evaluated the efficacy of mitotane treatment of 96 patients with adrenocortical carcinoma followed up in our department between 1959 and 1992. Complete tumour resection was the goal of the initial treatment. Mitotane treatment was classified according to serum trough concentrations on maintenance therapy: low ($<14\ mg\ l^{-1}$) or high ($\geq 14\ mg\ l^{-1}$). Total tumour resection was feasible in 47 patients (49%), and subtotal resection was performed in 37 patients (39%). Patients who underwent total tumour resection survived significantly longer than those who did not ($P<0.001$). Adjuvant mitotane therapy ($n = 11$) did not influence survival after total resection. Sixty-two patients were given mitotane treatment at some time during their illness, only 30 of whom reached high maintenance serum levels. Mitotane treatment with high serum levels had an independently favourable influence on patient survival, using univariate ($P<0.01$) and multivariate analysis ($P = 0.01$). Mitotane treatment resulting in low serum levels was tantamount to not giving mitotane at all. We conclude that mitotane treatment in adrenocortical carcinoma is effective only when high serum levels can be achieved.

Carcinoma of the adrenal cortex carries a poor prognosis (Macfarlane, 1958; Hutter & Kayhoe, 1966*a*; Venkatesh *et al.*, 1989; Luton *et al.*, 1990). According to recently reported clinical studies, only 20–25% of patients survive for more than 5 years after the diagnosis is made (Venkatesh *et al.*, 1989; Luton *et al.*, 1990). Early diagnosis and radical surgery offer the best hope for long-term survival. However, the diagnosis is frequently delayed and often only made at an advanced stage because of its rarity and the deep retroperitoneal localisation of the adrenal glands.

Another important factor contributing to the prognosis is the poor response to chemotherapeutic agents. Mitotane (*o,p'*-DDD) is considered to be the drug of choice for patients with inoperable, recurrent and metastatic disease (Samaan & Hickey, 1987). Increase in survival (Hutter & Kayhoe, 1966*b*; Lubitz *et al.*, 1973; Venkatesh *et al.*, 1989) and even long-term remission and cure of adrenocortical cancer (Becker & Schumacher, 1975; Jarabak & Rice, 1981) have been attributed to mitotane. Unfortunately, because of the rarity of the tumour, no randomised or controlled studies have been performed to assess mitotane's effect on patients' survival in adrenocortical carcinoma. However, a recently completed retrospective (multivariate) analysis of 105 patients with carcinoma of the adrenal cortex failed to document improved survival due to mitotane therapy (Luton *et al.*, 1990). In a retrospective study conducted in our hospital (van Slooten *et al.*, 1984) an improved survival was documented in 14 patients receiving mitotane therapy, when serum levels exceeded $14\ mg\ l^{-1}$.

The objective of this study was to evaluate the relevance of mitotane serum levels greater than $14\ mg\ l^{-1}$ in relation to other factors of possible beneficial influence on patient survival, in our extended series of 96 patients.

Correspondence: H.R. Haak, Department of Internal Medicine, Diaconessenhuis, PO Box 90.052, 5600 PD Eindhoven, The Netherlands.

Received 7 October 1993; and in revised form 6 January 1994.

### Patients and methods

### *Patients*

Ninety-six patients with adrenocortical carcinoma were evaluated and followed up in the Department of Endocrinology of the University Hospital of Leiden from 1959 to 1991. Follow-up for this report was closed on 1 January 1992. Patients underwent clinical, radiological and hormonal assessments. The 42 patients described in the series of van Slooten *et al.* (1984) are included in this report.

### *Definitions*

Clinical hormonal activity, assessed at the time of diagnosis, was considered to be present when medical history and physical examination were compatible with a diagnosis of Cushing's syndrome, Conn's syndrome, virilisation or feminisation.

Staging was based on clinical data and radiological studies at the time of diagnosis. Radiological assessments of the primary tumour were initially made with intravenous pyelogram and/or arteriography. Later patients were investigated with ultrasound, computerised tomography (CT) and some of them with magnetic resonance imaging (MRI). The radiological detection of metastases in the later patients could be done more reliably with ultrasound, CT or MRI scanning. Operative findings and pathological examination were included in the staging procedure. Local disease was defined as disease confined to the adrenal gland. Metastatic disease was defined as disease macroscopically extending beyond the limits of the adrenal gland into surrounding organs and tissues, or when distant metastases were present. Metastases becoming manifest within 3 months of surgery for the primary tumour were considered to be present at the time of surgery. Surgical resection of the tumour was considered total when local disease could be completely resected. In the case of post-operative macroscopically or microscopically residual tumour tissue, or in the case of metastatic disease, tumour resection was considered subtotal.

Evaluation of tumour response was based on the standard criteria of complete remission (no detectable disease), partial response (> 50% reduction in tumour mass for more than 1 month), stable disease and progressive disease (> 25% tumour increase). Survival periods were calculated from the time of diagnosis to the time of death.

### *Treatment*

*Surgery* Surgical excision of the primary tumour was always performed when the patients' clinical condition permitted it. Complete resection, metastases included, was the goal of surgery. When complete resection was not possible, maximal debulking of tumour load was pursued. Recurrent tumours were resected or debulked whenever possible.

*Mitotane* Mitotane therapy was started with 4–8 g per day given in four equal doses. In order to maximise intestinal absorption of the drug, treatment was started with mitotane given in chocolate, milkpowder or oil emulsion preparations (Moolenaar *et al.*, 1981). Maintenance therapy was continued in the form of tablets (Calbiochem or Bristol-Myers). Mitotane serum concentrations were determined according to the method described by Moolenaar *et al.* (1977). Blood for mitotane estimation was drawn at least 12 h after the last dose was taken. During treatment, serum levels were measured at least once a month. Maintenance serum levels were classified as low ($<14\ mg\ l^{-1}$) or high ($\geq 14\ mg\ l^{-1}$). The cut-off point was based on the empirical findings, reported by van Slooten *et al.* (1984), that seven of eight patients with tumour regression had mitotane serum levels above $14\ mg\ l^{-1}$, whereas 19 of 20 patients without an objective tumour regression had mitotane serum levels lower than $14\ mg\ l^{-1}$. Because of these findings, after 1981 a standard target mitotane serum trough level above $14\ mg\ l^{-1}$, and when possible over $20\ mg\ l^{-1}$, was aimed for. Mitotane therapy was continued for 2 years if resection was judged to be complete or for 1 year after apparent disappearance of the tumour. In addition to mitotane, all patients received hydrocortisone ($30-120\ mg\ day^{-1}$) or fludrocortisone acetate ($0.1-0.4\ mg\ day^{-1}$). Steroid replacement therapy started at the same time as the start of mitotane therapy when there was no, or only mild, clinical hormonal syndrome. In the case of an overt hormonal syndrome, steroid replacement was instituted when adrenal insufficiency became manifest, or was expected to occur. When necessary, the patients were treated with metoclopramide and loperamide to alleviate gastrointestinal side-effects.

*Other chemotherapy* Administration of other chemotherapeutic agents in addition to mitotane was considered in the presence of clinical and biochemical progression of adrenocortical carcinoma. In addition, chemotherapy was considered when a patient refused mitotane therapy or as judged by the attending physician. The CAP regimen (cyclophosphamide, doxorubicin and cisplatin) (van Slooten & Oosterom, 1983) was one of the therapeutic options. Streptozotocin (Erikson *et al.*, 1987) or cisplatin and etoposide (Johnson & Greco, 1986) were alternative therapies.

### *Statistical analysis*

Survival curves were calculated by the Kaplan–Meier method. The Lee–Desu statistic test was used for comparison of survival curves. A multivariate analysis according to the Cox proportional hazards model was performed to evaluate the independent effect of several factors on patient survival (see Results section) (Mathews & Farewell, 1985).

### Results

### *Clinical characteristics (Table I)*

The mean age of the patients at diagnosis was 44.4 years (s.d. 16.5 years). There were 56 female and 40 male patients. The left adrenal gland was involved in 51 (53%) patients and the right in 45 (47%) patients. Involvement was strictly unilateral.

Clinical hormonal syndromes were present in 39 (69%) of the female and 18 (45%) of the male patients. Thirty-nine patients (12 male and 27 female) had Cushing's syndrome and four patients had Conn's syndrome. Virilisation was present in one prepubertal boy and in 11 women; feminisation was observed in two men. At presentation, 22 men and 17 women demonstrated no abnormal endocrine features. Pain was the major non-endocrine symptom, and was documented in 45 (47%) of patients. A palpable abdominal mass was present in 28 (29%) patients.

Metastases were present in 38 patients at diagnosis. The liver was the most frequently affected site (26 patients), with the lungs being next in frequency (15 patients).

### *Treatment*

A flow chart of treatment is shown in Figure 1.

*Surgery* Eighty-four patients underwent surgery. Total tumour resection was possible in 47 patients. In eight of these patients the tumour capsule had ruptured during surgery. In

**Table I** Clinical details of 96 patients with adrenocortical carcinoma at the time of diagnosis

| | *Total (n = 96)* | | *Women (n = 56)* | | *Men (n = 40)* | |
|---|---|---|---|---|---|---|
| Age (years) | | | | | | |
| Mean (s.d.) | 44.4 (16.5) | | 43.3 (16.4) | | 45.9 (16.7) | |
| Range | 1–78 | | 1–71 | | 7–78 | |
| Tumour localisation | | | | | | |
| Left gland | 51 | | 28 | | 23 | |
| Right gland | 45 | | 28 | | 17 | |
| Clinical manifestations | | | | | | |
| Hormonal | 57 | | 39 | | 18 | |
| Cushing | | 39 | | 27 | | 12 |
| Virilisation | | 12 | | 11 | | 1 |
| Feminisation | | 2 | | 0 | | 2 |
| Conn | | 4 | | 1 | | 3 |
| Non-hormonal | 39 | | 17 | | 22 | |
| Other signs/symptoms | | | | | | |
| Pain | 45 | | 24 | | 21 | |
| Abdominal mass | 28 | | 17 | | 22 | |
| Metastases | 38 | | 21 | | 17 | |

**Figure 1** Flow chart of treatment in 96 patients with adrenocortical carcinoma. Eighty-four patients underwent surgery; in 47 of these patients a total tumour resection was possible. Sixty-two patients were treated with mitotane at some time during their illness. Thirty of these patients achieved high serum levels of the drug during maintenance therapy. Other chemotherapy was given to 21 patients. [a]No = no mitotane therapy; low = serum levels $<14 \text{ mg l}^{-1}$; high = serum levels $\geqslant 14 \text{ mg l}^{-1}$.

37 patients the tumour could not be resected completely. Twelve patients were judged to be inoperable.

Survival of patients with total tumour resection was significantly better than survival of patients undergoing only a subtotal tumour resection ($P<0.001$) (Figure 2). Five year survival after total resection was 49%, and after subtotal resection only 9%. Rupture of the tumour capsule during resection did not significantly influence survival ($P=0.38$). All patients with inoperable disease died within 18 months of diagnosis.

*Mitotane* Sixty-two patients received mitotane at some time during their illness between 1965 and 1991. The five patients diagnosed between 1959 and 1965 and not treated with mitotane had local disease. Maintenance therapy serum levels were found to be high (range $14-50 \text{ mg l}^{-1}$, 74% between 14 and $25 \text{ mg l}^{-1}$) in 30 patients and low (range $4-13 \text{ mg l}^{-1}$, 84% between 7 and $11 \text{ mg l}^{-1}$) in 32 others. The mitotane formulations from the two manufacturers were bioequivalent. No differences between serum levels reached depending on the brand used were observed.

Forty of the 62 patients who were treated with mitotane at some time during their illness received the drug early in the course of their illness. Twenty-nine of these 40 patients had measurable tumour size because they were not operated upon (five patients) or because they only underwent a subtotal resection (24 patients). A tumour response was only seen in six patients having high maintenance mitotane serum levels (Table II) ($P<0.001$, chi-square test). In three patients, a partial tumour response and in another three patients a complete remission lasting five, 69 and 190 (ongoing) months was observed. The high mitotane level group survival was significantly longer than that of the low-level group ($P<0.001$) (Figure 3).

One patient with a mitotane serum level less than $14 \text{ mg l}^{-1}$, and who was reported in the original series of van Slooten *et al.* (1984) to have a tumour response, did not meet the stricter criteria of WHO and set for the present study.

Mitotane therapy was given to 26 of the 38 patients who had a tumour recurrence. Four patients had also received a first course of mitotane treatment. Tumour response was

**Figure 2** Actuarial survival rates from time of diagnosis in 96 patients with adrenocortical carcinoma according to surgery. Total (T) = total tumour resection; subtotal (S) = subtotal tumour resection; no (N) = no tumour resection. T *vs* S, $P<0.001$; T *vs* N, $P<0.001$; S *vs* N, $P=0.05$.

again only observed in patients with a high maintenance mitotane level ($n=9$) (Table III) ($P<0.001$, chi-square test). Five of these patients had a complete remission lasting for 2–120 months at the time of reporting.

Mitotane therapy was given to 11 patients after apparently complete tumour resection. Six of these patients reached mitotane serum levels over $14 \text{ mg l}^{-1}$. Median survival of the 11 patients treated with adjuvant mitotane was 51 months. The median survival of the patients with complete tumour resection without adjuvant therapy was 61 months. The differences in survival and disease-free survival between these groups of patients were not significant.

*Mitotane side-effects* The main side-effects of mitotane were anorexia, nausea, vomiting, diarrhoea and CNS toxicity. Mitotane treatment had to be discontinued in ten patients because of side-effects. These were anorexia and nausea in eight patients and neuropsychiatric symptoms in two patients. Nine of the ten patients stopping treatment because of side-effects never achieved high mitotane serum levels.

**Figure 3** Actuarial survival rates from time of diagnosis in 29 patients with evaluable tumour (no operation, $n = 5$; subtotal operation, $n = 24$) according to serum levels of mitotane (mitotane therapy given early in the course of their disease). High (H) = serum levels $> 14\ mg\ l^{-1}$; low (L) = serum levels $< 14\ mg\ l^{-1}$. H *vs* L, $P < 0.001$.

**Table II** Response[a] of the primary tumour to mitotane therapy in relation to mitotane serum levels ($n = 29$)

| | $< 14\ mg\ l^{-1}$ | $\geqslant 14\ mg\ l^{-1}$ |
|---|---|---|
| Response | 0 | 6 |
| No response | 15 | 8 |

$P = 0.004$ (chi-square test). [a]Response includes complete remission and partial response.

**Table III** Response[a] of recurrent tumour to mitotane therapy in relation to mitotane serum levels ($n = 23$)

| | $< 14\ mg\ l^{-1}$ | $\geqslant 14\ mg\ l^{-1}$ |
|---|---|---|
| Response | 0 | 9 |
| No response | 10 | 4 |

$P = 0.0008$ (chi-square test). [a]Response includes complete remission and partial response.

Gastrointestinal manifestations were present early in the course of treatment but in most patients could be controlled well enough not to require discontinuation of the drug. Mitotane serum levels associated with the gastrointestinal side-effects were all above $5\ mg\ l^{-1}$. CNS toxicity was seen especially with high mitotane serum levels, with cerebellar ataxia being the most common manifestation. Neuropsychological impairment was found during therapy. Deterioration varied individually from mild to severe, but always occurred when mitotane serum level exceeded $15\ mg\ l^{-1}$. CNS toxicity reversed completely after drug withdrawal. A prolonged bleeding time was encountered in 90% of patients tested for platelet function ($n = 10$). Less frequent side-effects observed were rash (2–6 weeks after starting therapy) and a mild leucopenia.

*Other therapy* Twenty-one patients received one or two different chemotherapeutic regimens, apart from mitotane, at some time during their illness. Ten patients were treated early in the course of the disease and 11 during recurrence of the disease. The CAP regimen was administered to 12 patients, in two patients after unsuccessful mitotane treatment. Two patients achieved partial remissions lasting 18 and 23 months.

Seven patients received streptozotocin after tumour progression under mitotane, all without objective tumour response. Three patients received the cisplatin–etoposide regimen without tumour response.

*Multivariate analysis of survival* Using the Cox proportional hazards model, we evaluated the influence of the following variables on patient survival: (a) outcome of first surgery (no resection, subtotal or total resection); (b) mitotane treatment at some time during illness (no mitotane, low and high mitotane levels); (c) other chemotherapy at some time during illness (no, yes); (d) age at diagnosis ($\leqslant 40$ years, $> 40$ years); (e) year of diagnosis ($< 1980$, $\geqslant 1980$); (f) sex (male, female); and (g) clinical features of hormonal dysfunction at presentation (no, yes). In all patients stepwise analysis of these variables showed that total resection at first surgery ($P < 0.001$), mitotane treatment at some time during illness with high serum levels ($P = 0.01$) and female sex ($P = 0.03$) had independently a favourable influence on cumulative survival.

Stepwise analysis on the 49 patients who could not be operated upon ($n = 12$) or who had a subtotal tumour resection ($n = 37$) showed an independent, favourable influence on survival when mitotane treatment given at some time during illness resulted in high maintenance serum levels ($P < 0.001$) and when treatment with other chemotherapy was undertaken ($P = 0.005$).

### Discussion

The best chance of survival for a patient with adrenocortical carcinoma is when a complete tumour resection can be performed. Although excision of metastases could not cure our patients, we feel that debulking of tumour mass must be maximised since chemotherapy is considered to be more effective when tumour load is low (Harris & Mastrangelo, 1991).

In our series, more than half of the patients with a seemingly complete tumour resection died of their disease within 5 years of surgery. It has been reported that local recurrence or metastases occur in 80% of the patients after radical surgery (Lipsett *et al.*, 1963; Bertagna & Orth, 1981). Spillage of tumour during operation may be the cause of tumour recurrence. However, in our series, rupture of the tumour capsule during surgery was not associated with shorter survival.

Our findings suggest a significant favourable effect of mitotane treatment on patient survival in adrenocortical carcinoma. Other authors have previously suggested a similar favourable effect (Hutter & Kayhoe, 1966*b*; Lubitz *et al.*, 1973; Venkatesh *et al.*, 1989), but to our knowledge this has never been well documented. Moreover, in a large retrospective study Luton *et al.* (1990) could not find an independent beneficial effect resulting from mitotane therapy. Apart from the effectiveness of mitotane treatment, there are no major differences between our study and that of Luton *et al.* The discrepancy in findings between the two studies may lie in the different dosages of the drug used (or serum concentrations reached), as was suggested by van Slooten *et al.* in 1984 (Haak *et al.*, 1990). In the present study, an objective tumour response, according to the strict WHO criteria, was found in 15 of the 27 evaluable patients with levels above $14\ mg\ l^{-1}$. Continuous serum levels under $14\ mg\ l^{-1}$ resulted in no tumour response, and was tantamount to not giving mitotane at all.

The differential effect of sex on patient survival observed in our series is difficult to explain. The longer survival observed in patients who received other chemotherapy cannot be explained by objective tumour responses. It is possible that chemotherapy retarded tumour progression in our patients, resulting in a better survival. However, a bias may well have occurred in selecting patients for treatment with chemotherapy.

Despite all possible reservations against a retrospective study, we feel that our results prove the efficacy of mitotane in adrenocortical carcinoma. We have also clearly shown that a patient with adrenocortical carcinoma may only benefit from mitotane therapy when high serum levels of the drug can be reached.

An important reason for not reaching therapeutic levels may be undertreatment with steroids, resulting in signs and symptoms of adrenocortical insufficiency. Because of increased serum steroid-binding capacity during mitotane therapy, gluco- and mineralocorticoids must be supplied in higher than normal amounts (van Seters & Moolenaar, 1991). A physician not aware of this will be more easily inclined to lower mitotane dosage or even to stop the drug. Of course, primary resistance of the tumour to mitotane and rapid progression of the disease may be a major factor in failure of the therapy.

Adjuvant mitotane therapy has been advocated after complete tumour resection (Venkatesh *et al.*, 1989; Luton *et al.*, 1990). In our study, patients who were treated adjuvantly with mitotane did not do better than those who did not receive it. On theoretical grounds, it is advisable to treat patients with an active drug when tumour load is low (Harris & Mastrangelo, 1991). More than half of the patients develop recurrent disease after total tumour resection and must thus have residual tumour tissue. Since recurrence cannot be predicted, we feel that adjuvant therapy must be considered in all patients after total tumour resection. However, the majority of patients will not benefit from this treatment, for example when the carcinoma has been confidently completely resected, or when the carcinoma is unresponsive to mitotane. As the treatment is a burden to most patients, we would not advocate adjuvant mitotane treatment in patients with adrenocortical carcinoma after complete tumour resection.

We conclude that surgery offers the best hope for long-term survival for patients with adrenocortical carcinoma. Mitotane treatment for patients with inoperable, recurrent, and/or metastatic adrenocortical carcinoma is effective, provided that serum levels of the drug are maintained above 14 mg $l^{-1}$.

We thank L. Cobben for his assistance in collecting the data and Dr N.A.T. Hamdy and Professor Dr F.J. Cleton for critical review of the manuscript.

### References

BECKER, D. & SCHUMACHER, O.P. (1975). O,p'DDD therapy in invasive adrenocortical carcinoma. *Ann. Intern. Med.*, **82**, 677–679.

BERTAGNA, C. & ORTH, D.N. (1981). Clinical and laboratory findings and results of therapy in 58 patients with adrenocortical tumors admitted to a single medical center (1951 to 1978). *Am. J. Med.*, **71**, 855–875.

ERIKSON, B., OBERG, K., CURSTEDT, T., HEMMINGSSON, A., JOHANSSON, H., LINDH, E., LINDGREN, P.G., THUOMAS, K.A., WILANDER, E. & AKERSTROM, G. (1987). Treatment of hormone producing adrenocortical cancer with o,p'DDD and streptozotocin. *Cancer*, **58**, 1398–1403.

HAAK, H.R., VAN SETERS, A.P. & MOOLENAAR, A.J. (1990). Mitotane therapy of adrenocortical carcinoma. *N. Engl. J. Med.*, **323**, 758.

HARRIS, D.T. & MASTRANGELO, M.J. (1991). Theory and application of early systemic therapy. *Semin. Oncol.*, 493–503.

HUTTER, Jr, A.M. & KAYHOE, D.E. (1966*a*). Adrenal cortical carcinoma: clinical features of 138 patients. *Am. J. Med.*, **41**, 572–580.

HUTTER, A.M. & KAYHOE, D.E. (1966*b*). Adrenal cortical carcinoma: results of treatment with o,p'DDD in 138 patients. *Am. J. Med.*, **41**, 581–592.

JARABAK, J. & RICE, K. (1981). Metastatic adrenal cortical carcinoma: prolonged regression with mitotane therapy. *JAMA*, **246**, 1706–1707.

JOHNSON, D.H. & GRECO, F.A. (1986). Treatment of metastatic adrenal cortical carcinoma with cisplatin and etoposide (VP-16). *Cancer*, **58**, 2198–2202.

LIPSETT, M.B., HERTZ, R. & ROSS, G.T. (1963). Clinical and pathophysiologic aspects of adrenocortical carcinoma. *Am. J. Med.*, **35**, 374–383.

LUBITZ, J.A., FREEMAN, L. & OKUN, R. (1973). Mitotane use in inoperable adrenal cortical carcinoma. *JAMA*, **223**, 1109–1112.

LUTON, J.P., CERDAS, S., BILLAUD, L., THOMAS, G., GUILHAUME, B., BERTAGNA, X., LAUDAT, M.H., LOUVEL, A., CHAPUIS, Y., BLONDEAU, PH., BONNIN, A. & BRICAIRE, H. (1990). Clinical features of adrenocortical carcinoma, prognostic factors, and the effect of mitotane therapy. *N. Engl. J. Med.*, **322**, 1195–1201.

MACFARLANCE, D.A. (1958). Cancer of the adrenal cortex: the natural history, prognosis and treatment in a study of fifty-five cases. *Ann. R. Coll. Surg. Engl.*, **23**, 155–186.

MATHEWS, D.E. & FAREWELL, V. (1985). *Using and Understanding Medical Statistics*. Karger: Basle.

MOOLENAAR, A.J., NIEWINT, J.W.M. & OEI, I.T. (1977). Estimation of o,p'-DDD in plasma by gas-liquid chromatography. *Clin. Chim. Acta.*, **76**, 213–218.

MOOLENAAR, A.J., VAN SLOOTEN, H., VAN SETERS, A.P. & SMEENK, D. (1981). Blood levels of o,p'-DDD following administration in various vehicles after a single dose and during long-term treatment. *Cancer Chemother. Pharmacol.*, **7**, 51–54.

SAMAAN, N.A. & HICKEY, R.C. (1987). Adrenal cortical carcinoma. *Semin. Oncol.*, **14**, 292–296.

VAN SETERS, A.P. & MOOLENAAR, A.J. (1991). Mitotane increases the blood level of hormone-binding proteins. *Acta Endocrinol.*, **124**, 526–533.

VAN SLOOTEN, H. & OOSTEROM, A.T. (1983). CAP (cyclophosphamide, doxorubicin, and cisplatin) regimen in adrenal cortical carcinoma. *Cancer Treat. Rep.*, **67**, 377–379.

VAN SLOOTEN, H., MOOLENAAR, A.J., VAN SETERS, A.P. & SMEENK, D. (1984). The treatment of adrenocortical carcinoma with o,p'-DDD: prognostic implications of serum level monitoring. *Eur. J. Clin. Oncol.*, **20**, 47–53.

VENKATESH, S., HICKEY, R.C., SELLIN, R.V., FERNANDEZ, J.F., SAMAAN, N.A. (1989). Adrenal cortical carcinoma. *Cancer*, **64**, 765–769.


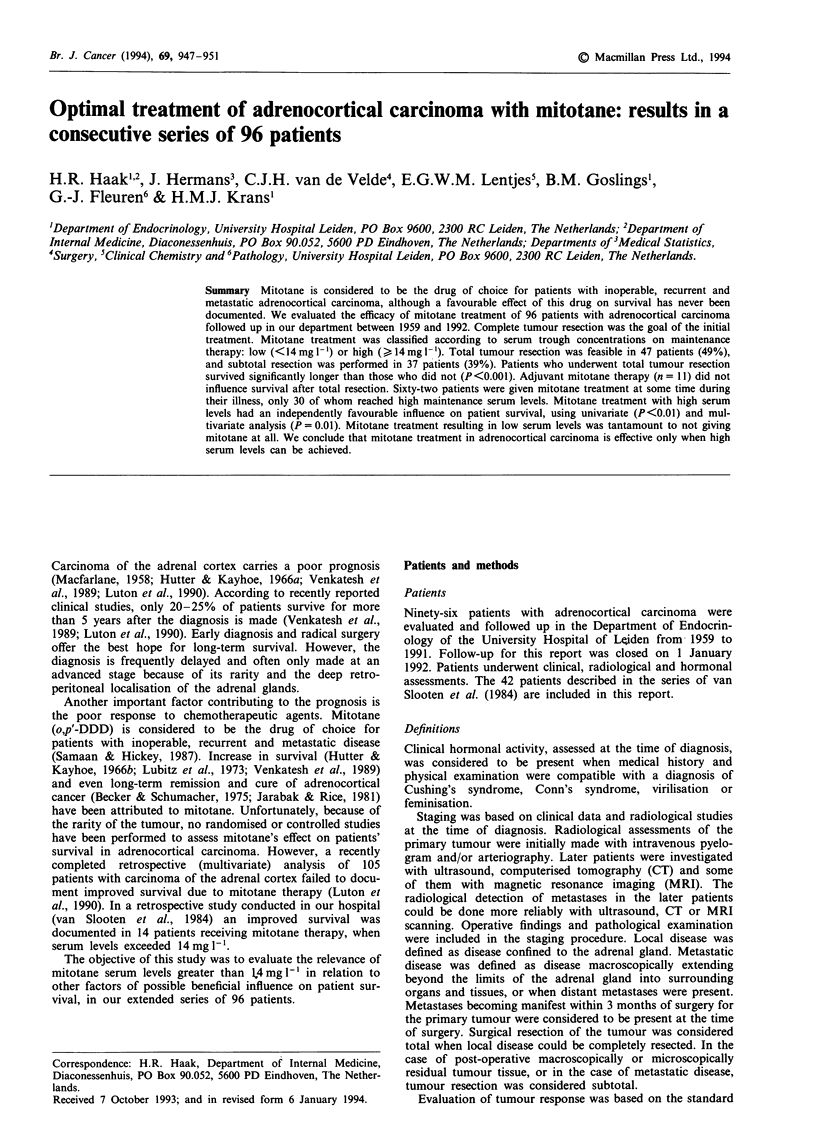

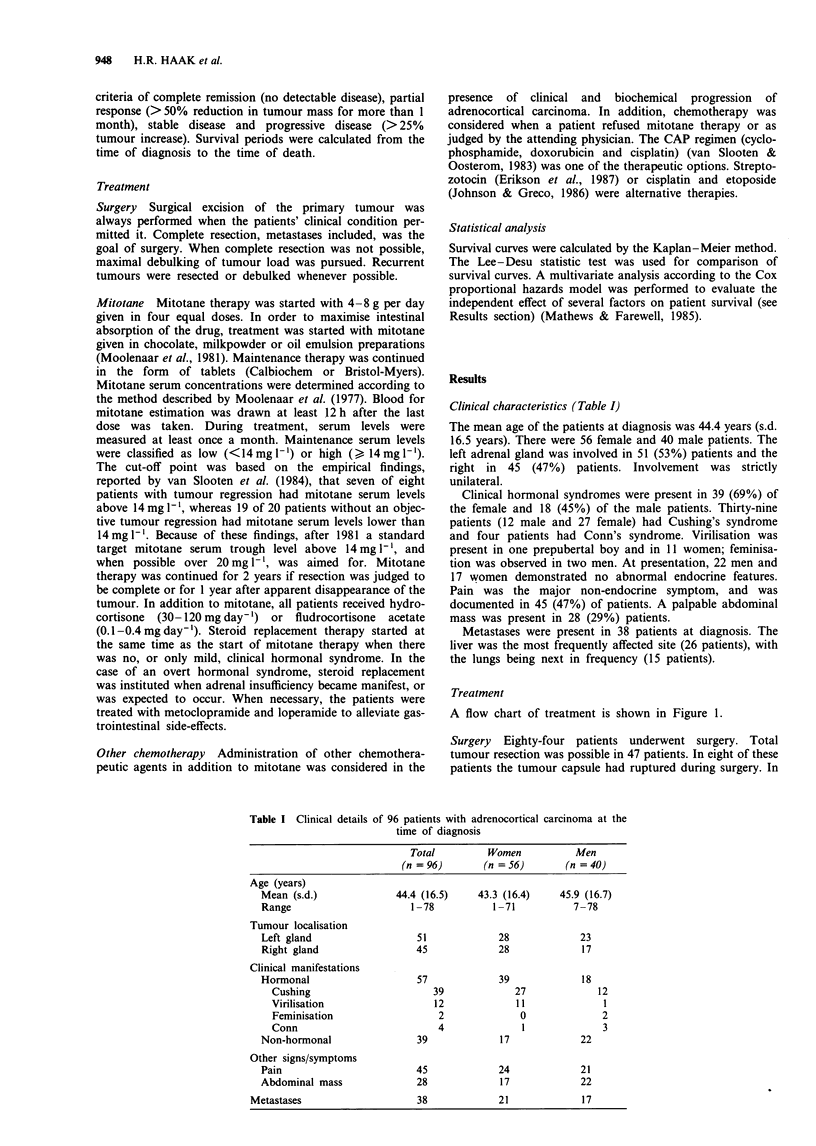

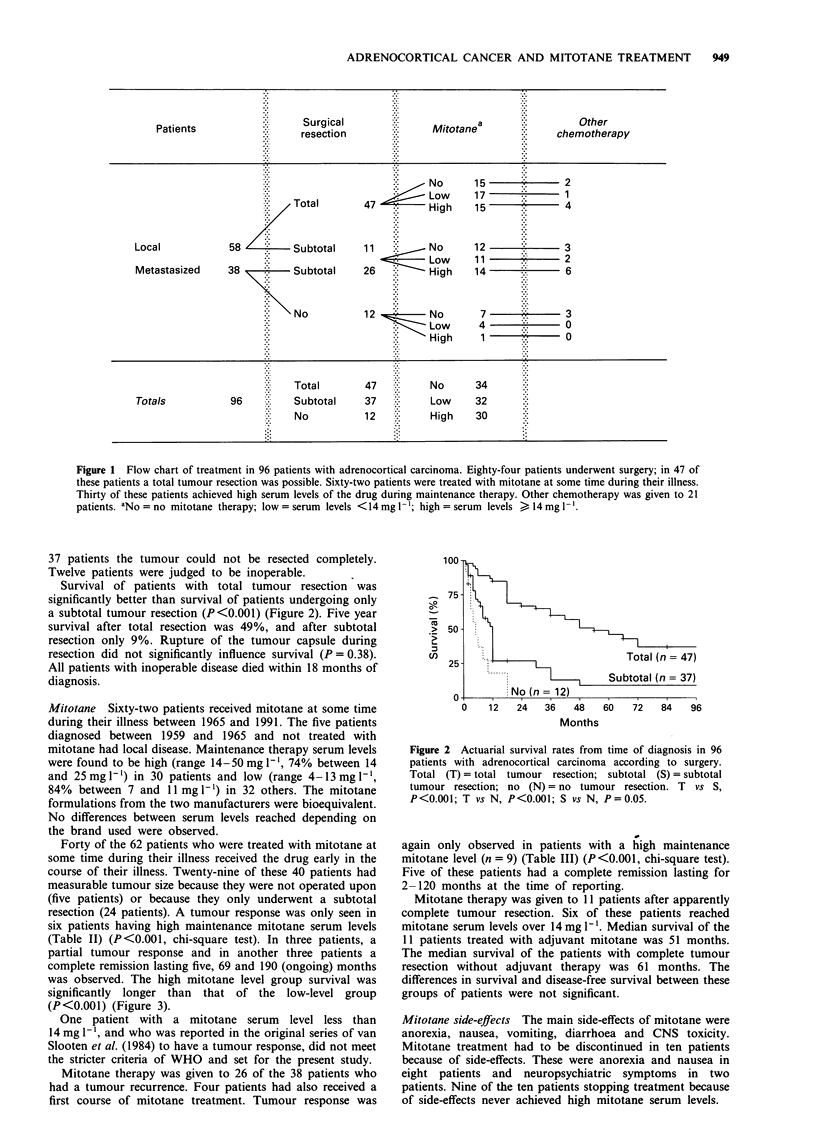

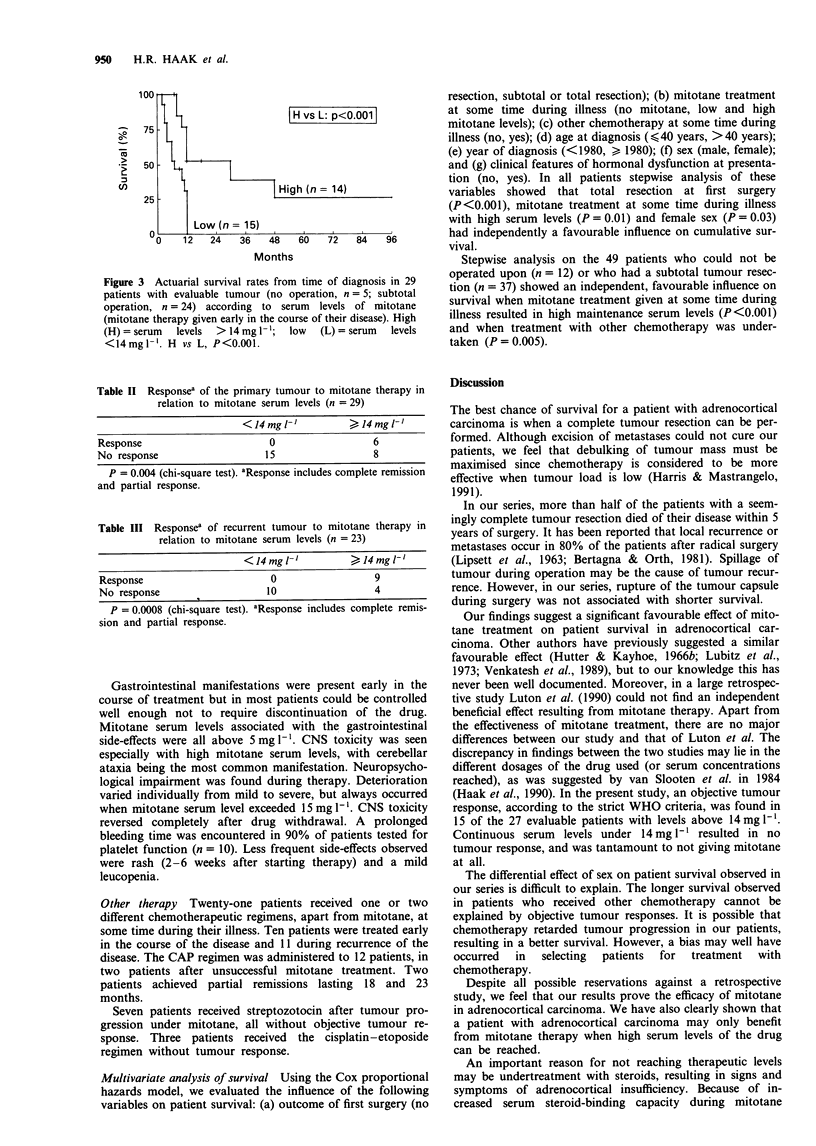

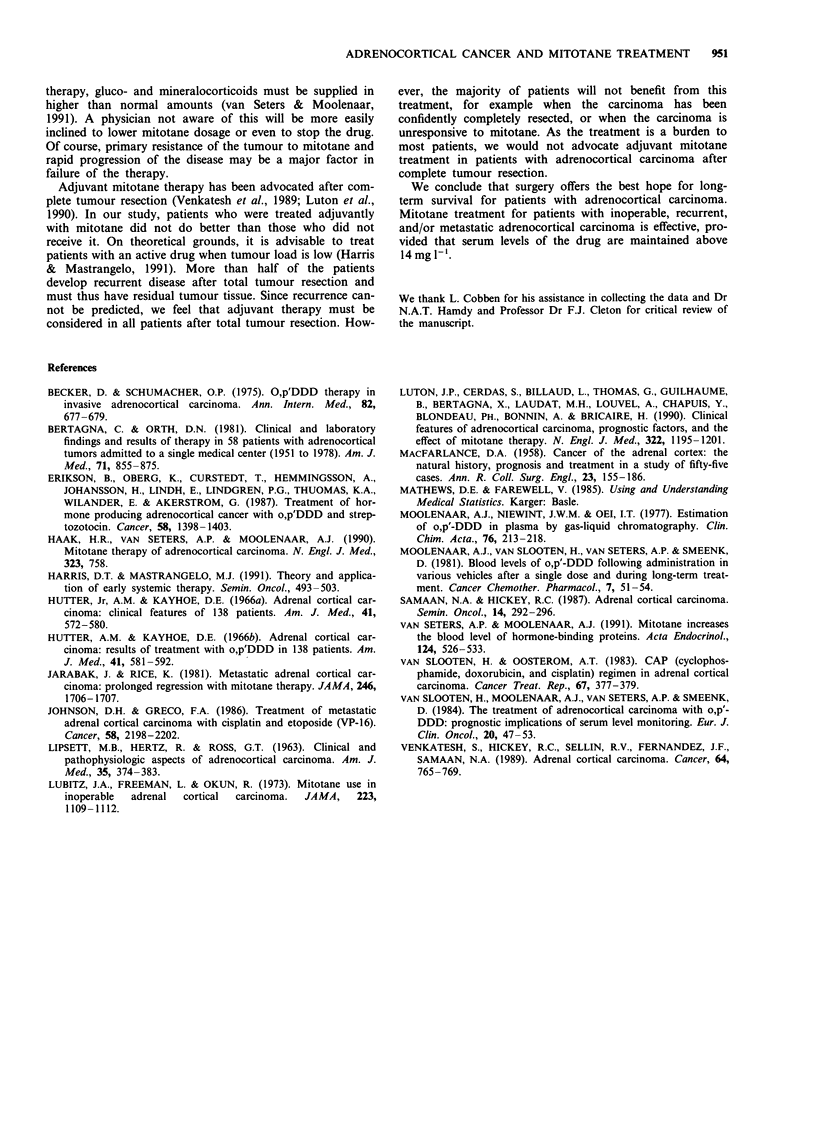


## References

[OCR_00585] Becker D., Schumacher O. P. (1975). o,p'DDD therapy in invasive adrenocortical carcinoma.. Ann Intern Med.

[OCR_00590] Bertagna C., Orth D. N. (1981). Clinical and laboratory findings and results of therapy in 58 patients with adrenocortical tumors admitted to a single medical center (1951 to 1978).. Am J Med.

[OCR_00596] Eriksson B., Oberg K., Curstedt T., Hemmingsson A., Johansson H., Lindh E., Lindgren P. G., Thuomas K. A., Wilander E., Akerström G. (1987). Treatment of hormone-producing adrenocortical cancer with o,p'DDD and streptozocin.. Cancer.

[OCR_00603] Haak H. R., van Seters A. P., Moolenaar A. J. (1990). Mitotane therapy of adrenocortical carcinoma.. N Engl J Med.

[OCR_00608] Harris D. T., Mastrangelo M. J. (1991). Theory and application of early systemic therapy.. Semin Oncol.

[OCR_00612] Hutter A. M., Kayhoe D. E. (1966). Adrenal cortical carcinoma. Clinical features of 138 patients.. Am J Med.

[OCR_00617] Hutter A. M., Kayhoe D. E. (1966). Adrenal cortical carcinoma. Results of treatment with o,p'DDD in 138 patients.. Am J Med.

[OCR_00622] Jarabak J., Rice K. (1981). Metastatic adrenal cortical carcinoma. Prolonged regression with mitotane therapy.. JAMA.

[OCR_00627] Johnson D. H., Greco F. A. (1986). Treatment of metastatic adrenal cortical carcinoma with cisplatin and etoposide (VP-16).. Cancer.

[OCR_00632] LIPSETT M. B., HERTZ R., ROSS G. T. (1963). CLINICAL AND PATHOPHYSIOLOGIC ASPECTS OF ADRENOCORTICAL CARCINOMA.. Am J Med.

[OCR_00637] Lubitz J. A., Freeman L., Okun R. (1973). Mitotane use in inoperable adrenal cortical carcinoma.. JAMA.

[OCR_00642] Luton J. P., Cerdas S., Billaud L., Thomas G., Guilhaume B., Bertagna X., Laudat M. H., Louvel A., Chapuis Y., Blondeau P. (1990). Clinical features of adrenocortical carcinoma, prognostic factors, and the effect of mitotane therapy.. N Engl J Med.

[OCR_00648] MACFARLANE D. A. (1958). Cancer of the adrenal cortex; the natural history, prognosis and treatment in a study of fifty-five cases.. Ann R Coll Surg Engl.

[OCR_00657] Moolenaar A. J., Niewint J. W., Oei I. T. (1977). Estimation of o,p'-DDD in plasma by gas-liquid chromatography.. Clin Chim Acta.

[OCR_00662] Moolenaar A. J., van Slooten H., van Seters A. P., Smeenk D. (1981). Blood levels of o,p'-DDD following administration in various vehicles after a single dose and during long-term treatment.. Cancer Chemother Pharmacol.

[OCR_00668] Samaan N. A., Hickey R. C. (1987). Adrenal cortical carcinoma.. Semin Oncol.

[OCR_00688] Venkatesh S., Hickey R. C., Sellin R. V., Fernandez J. F., Samaan N. A. (1989). Adrenal cortical carcinoma.. Cancer.

[OCR_00672] van Seters A. P., Moolenaar A. J. (1991). Mitotane increases the blood levels of hormone-binding proteins.. Acta Endocrinol (Copenh).

[OCR_00682] van Slooten H., Moolenaar A. J., van Seters A. P., Smeenk D. (1984). The treatment of adrenocortical carcinoma with o,p'-DDD: prognostic implications of serum level monitoring.. Eur J Cancer Clin Oncol.

[OCR_00677] van Slooten H., van Oosterom A. T. (1983). CAP (cyclophosphamide, doxorubicin, and cisplatin) regimen in adrenal cortical carcinoma.. Cancer Treat Rep.

